# Author Correction: Functional analysis of the Aspergillus fumigatus kinome identifies a druggable DYRK kinase that regulates septal plugging

**DOI:** 10.1038/s41467-024-50129-y

**Published:** 2024-08-20

**Authors:** Norman van Rhijn, Can Zhao, Narjes Al-Furaiji, Isabelle S. R. Storer, Clara Valero, Sara Gago, Harry Chown, Clara Baldin, Rachael-Fortune Grant, Hajer Bin Shuraym, Lia Ivanova, Olaf Kniemeyer, Thomas Krüger, Elaine Bignell, Gustavo H. Goldman, Jorge Amich, Daniela Delneri, Paul Bowyer, Axel A. Brakhage, Hubertus Haas, Michael J. Bromley

**Affiliations:** 1 Manchester Fungal Infection Group, Division of Evolution, Infection and Genomic Sciences, Faculty of Biology, Medicine and Health, University of Manchester, Manchester, UK; 2 Department of Pharmacology, College of Medicine, University of Kerbala, Kerbala, Iraq; 3 Division of Molecular Biology, Biocenter, Innsbruck Medical University, Innsbruck, Austria; 4 Department of Clinical Laboratory Sciences, College of Applied Medical Sciences, King Saud Bin Abdulaziz University for Health Sciences, 11481 Riyadh, Saudi Arabia; 5 Department of Molecular and Applied Microbiology, Leibniz Institute for Natural Product Research and Infection Biology (Leibniz-HKI), Jena, Germany; 6 MRC Centre for Medical Mycology, University of Exeter, Stocker Road, Exeter, EX4 4QD UK; 7 Faculdade de Ciências Farmacêuticas de Ribeirão Preto, Universidade de São Paulo, Ribeirão Preto, São Paulo, Brazil; 8 Mycology Reference Laboratory (Laboratorio de Referencia e Investigación en Micología [LRIM]), National Centre for Microbiology, Instituto de Salud Carlos III (ISCIII), Majadahonda, Madrid, Spain; 9 Division of Evolution, Infection and Genomic Sciences, Faculty of Biology, Medicine and Health, University of Manchester, Manchester, UK; 10 Department of Microbiology and Molecular Biology, Institute of Microbiology, Friedrich Schiller University, Jena, Germany

**Keywords:** Fungal genetics, Antifungal agents, Fungal biology

Correction to: *Nature Communications* 10.1038/s41467-024-48592-8, published online 11 June 2024

In this article the author’s name Narjes Al-Furaiji was incorrectly written as Narjes Al-Furaji. Narjes Al-Furaiji should have also been denoted as an equally contributing author. The funding from The Ministry of Higher Education and Scientific Research (MOHESR), Iraq. to N.A.F and M.B. was omitted. The original article has been corrected.

