# Supplementary material for: Functional analysis of the Aspergillus fumigatus kinome identifies a druggable DYRK kinase that regulates septal plugging
Source: Nat Commun. 2024 Jun 11;15:4984. doi: 10.1038/s41467-024-48592-8 (PMC11166925; doi:10.1038/s41467-024-48592-8)
Supplement: Supplementary file 1 — Supplementary Information [file 41467_2024_48592_MOESM1_ESM.pdf]

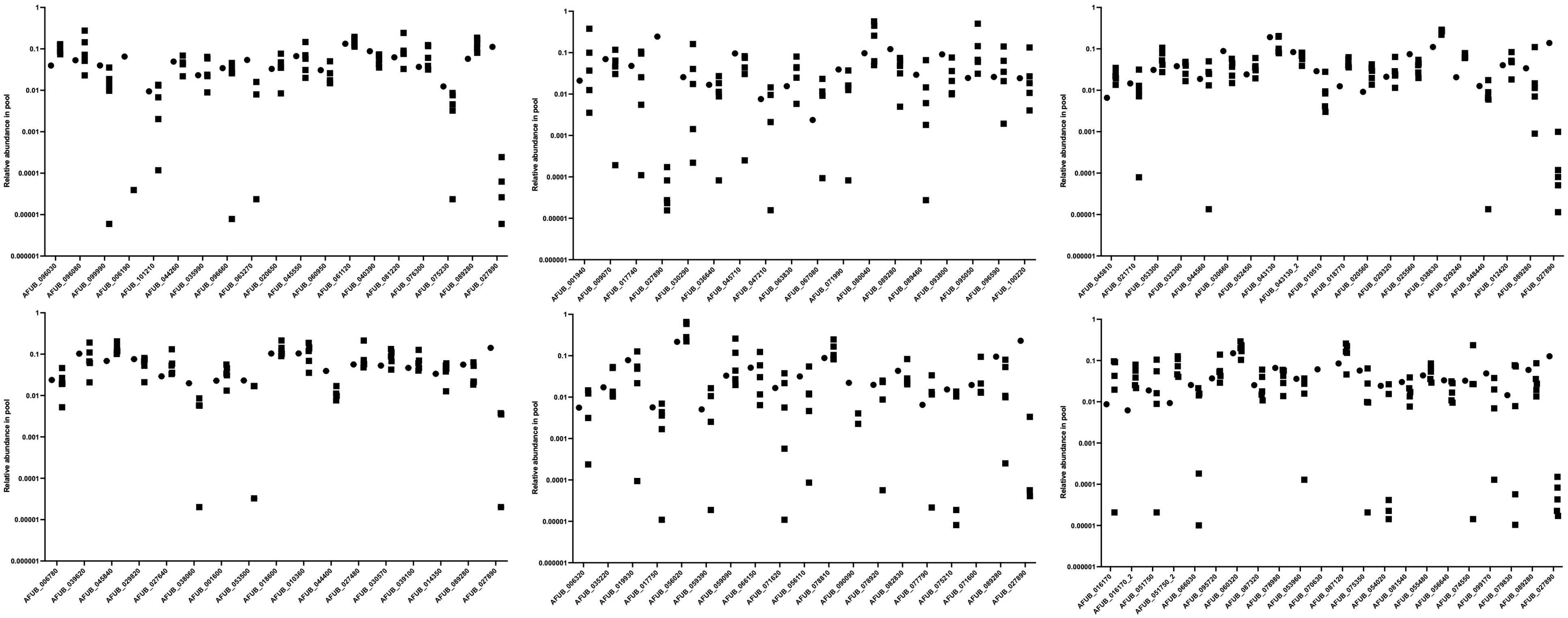

Supplemental Figure 1: Fitness of protein kinase null mutants in a leukopenic mouse model. Six pools from the protein kinase null mutant collection were used to inoculate mice rendered leukopenic (by cyclophosphamide (IP) and triamcinolone (SC)). The relative abundance of each strain in the pools are shown as filled circles while the abundance of the strain in lungs isolated from infected mice (n=5) are shown as filled squares. Data points with values of zero are not shown.

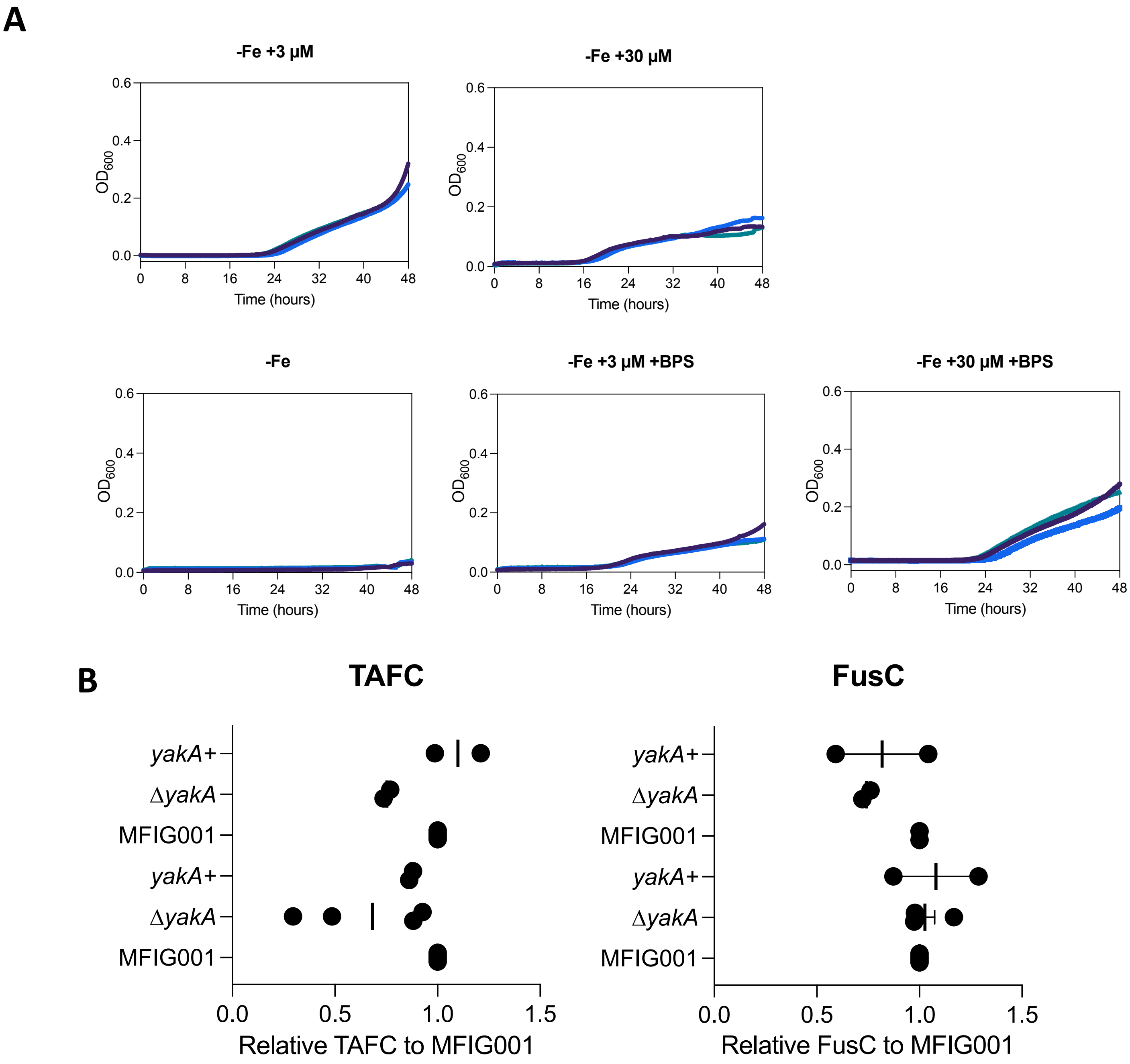

Supplemental Figure 2: Growth in static liquid culture of ΔyakA and siderophore production. A) Growth of MFIG001, Δ*yakA* and *yakA*+ was assessed over 48 hours in microwell format in AMM with different concentrations of iron and addition of BPS, measuring optical density (OD_{600}) every 10 minutes. No significant difference between strains was observed. B) Siderophore production (triacetylfusarinine C [TAFC] and Fusarinine C [FusC]) in MFIG001 and Δ*yakA* was measured in AMM iron replete and limiting conditions. No significant differences were observed between WT and Δ*yakA*.

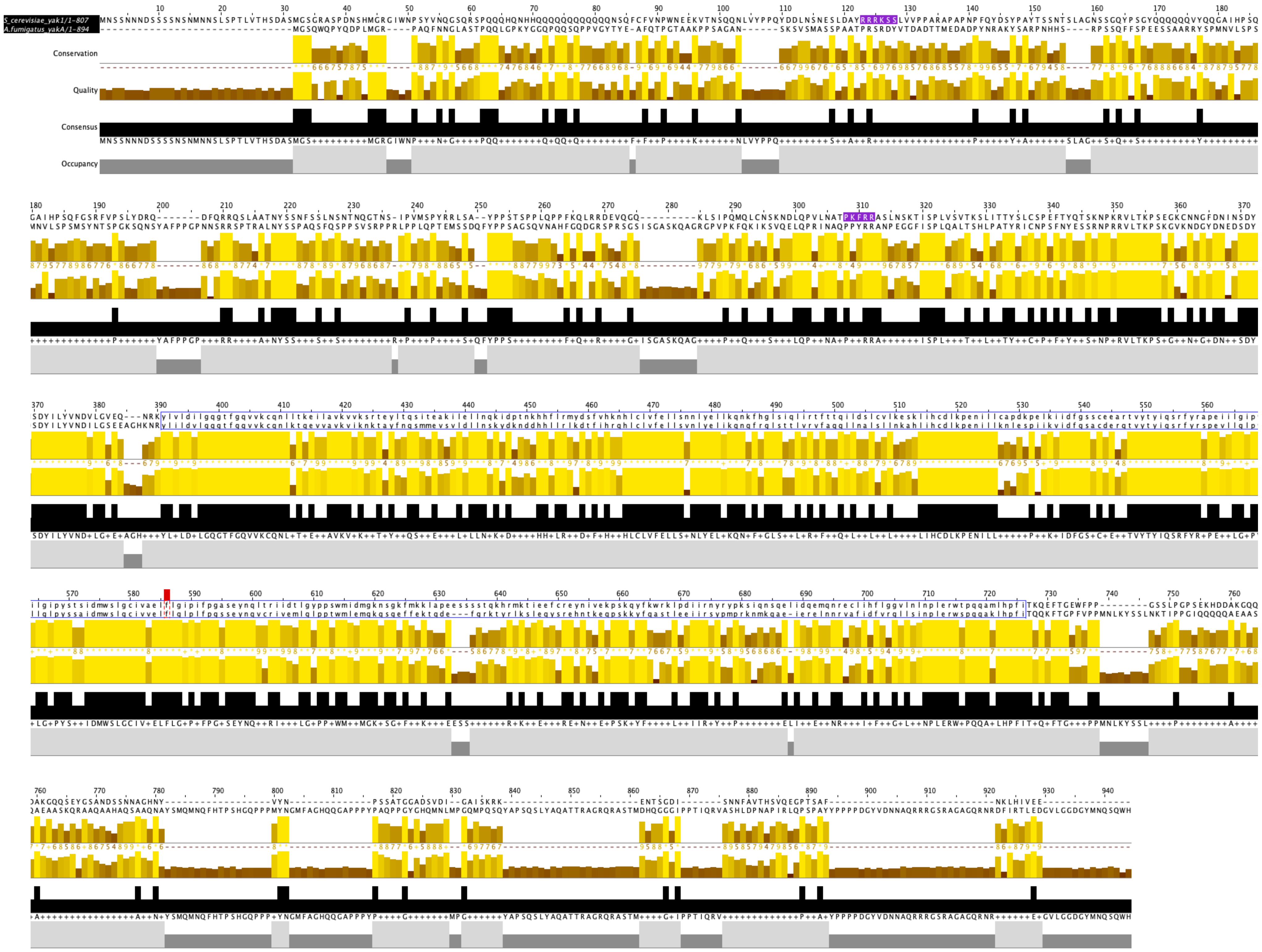

Supplemental Figure 3: Alignment of *S. cerevisiae* Yak1 and *A. fumigatus* YakA.. Alignment between Yak1 and YakA was performed using Muscle (standard parameters) and visualised in Jalview. Conservation score, quality, consensus and occupancy is shown. The NLS sequences are highlighted in purple and the kinase domain in lowercase, surrounded by a blue box in the sequence.

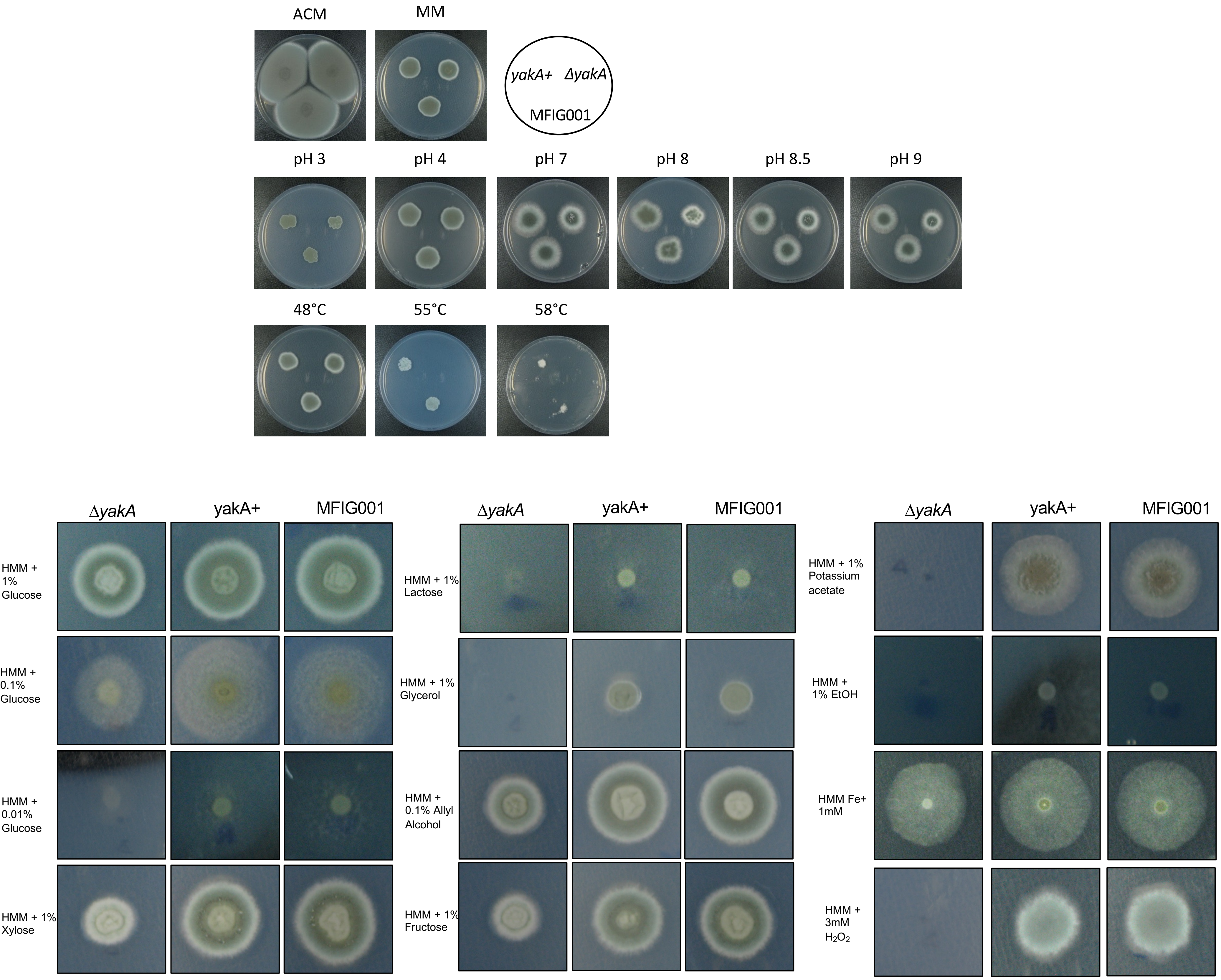

Supplemental Figure 4: Phenotypic characterisation of Δ*yakA*. MFIG001, Δ*yakA* and the reconstituted isolate *yakA*+ were spot inoculated (10^3 spores) on a range of different media and incubated at 37°C for 72 hours.

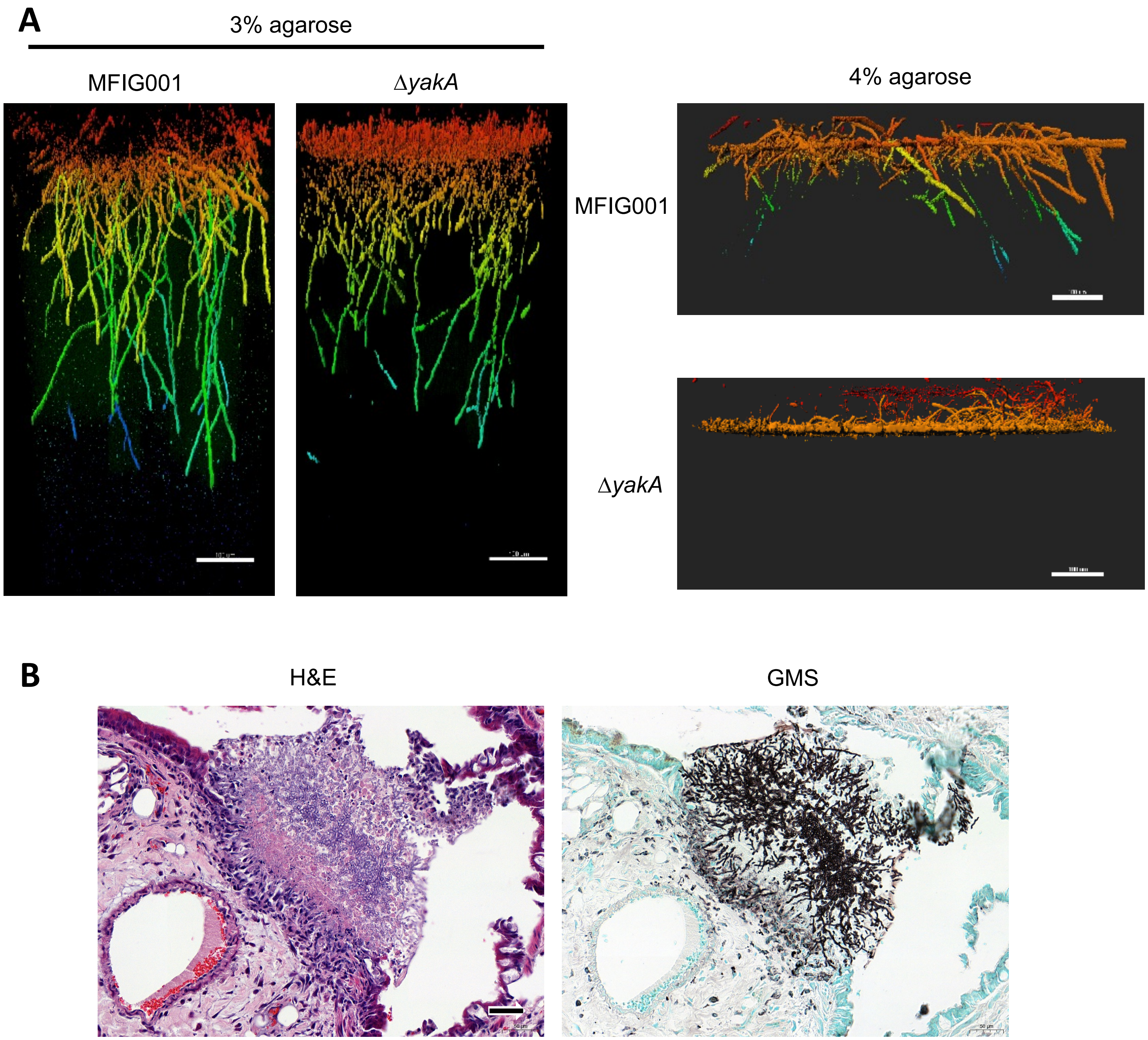

Supplemental Figure 5: Penetration of Δ*yakA*. A) 3D imaging of MFIG001 and Δ*yakA* after 4 hours in 3% and 4% agarose solid medium. Hyphal reconstruction was made in IMARIS and depth of penetration of individual hyphae is shown in colour. Scalebar = 100 μm B) A representative image of Δ*yakA* in major airways of leukopenic mouse lungs. H&E and GMS staining was performed on subsequent sections. Scale bar = 50 μm.

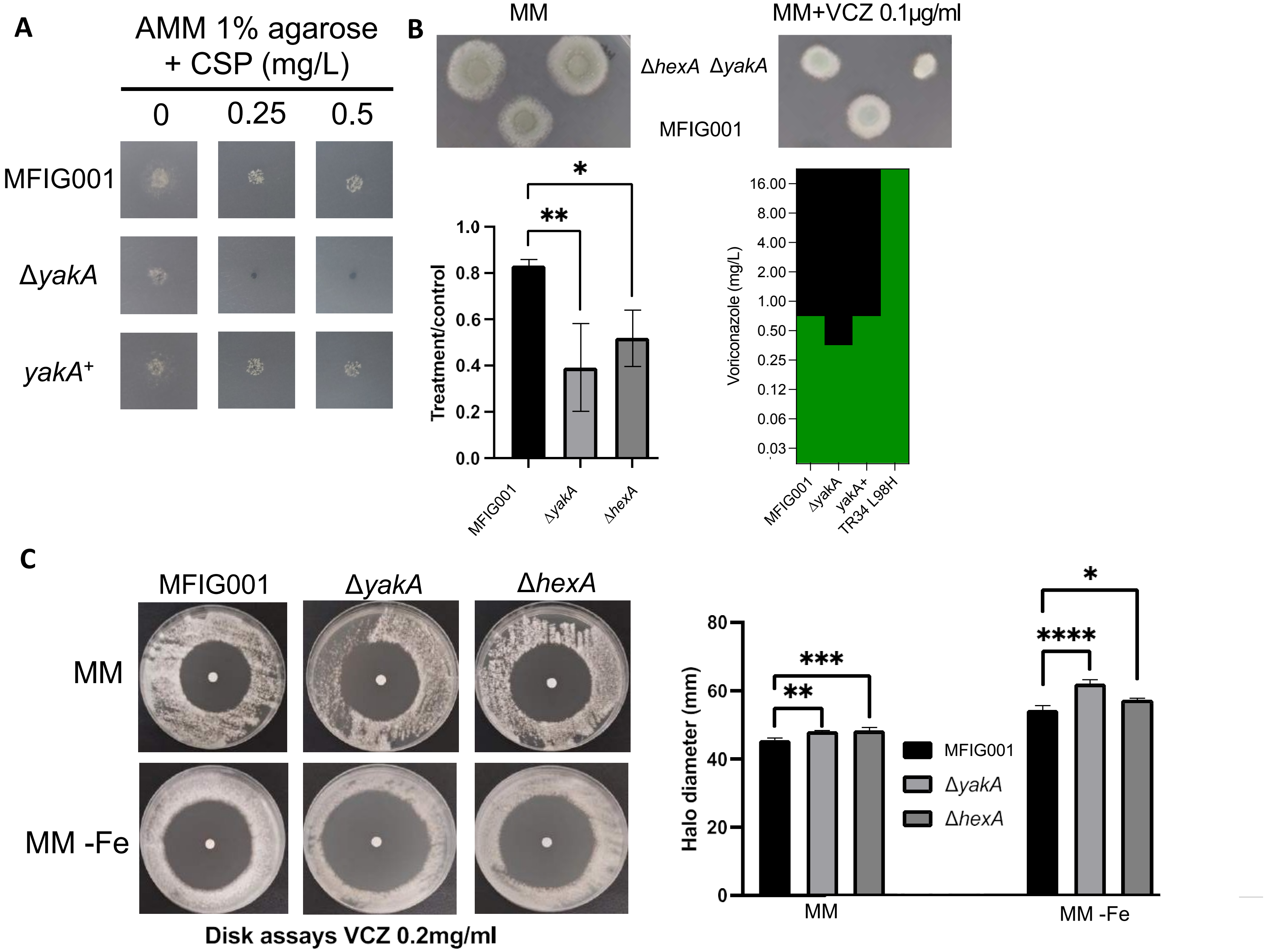

Supplemental Figure 6: **Phenotypic analysis of Δ*yakA* and Δ*hexA*. A.** 100 spores per strain were spotted on AMM, with or without caspofungin and incubated at 37 Celsius for 48 hours. B. MFIG001, Δ*yakA* and Δ*hexA* on AMM solid plates under voriconazole exposure. 100 spores per strain were spotted and incubated for 3 days at 37 Celsius (n=3). Quantification of radial growth is shown and statistical difference was assessed via one-way ANOVA. MIC was determined by standard EUCAST methodology and read after 48 hours via microscopic evaluation. Green is classed as growth and black as no growth at given voriconazole concentration. C. Disc assays to show susceptibility to voriconazole on solid AMM plates (+Fe or -Fe). Plates were incubated at 37 Celsius for 48 hours, followed by measuring of the halo (n=3). Representative images are shown and quantification. Statistical difference was assessed by one-way ANOVA (* p<0.05, ** p<0.01, *** p<0.001).

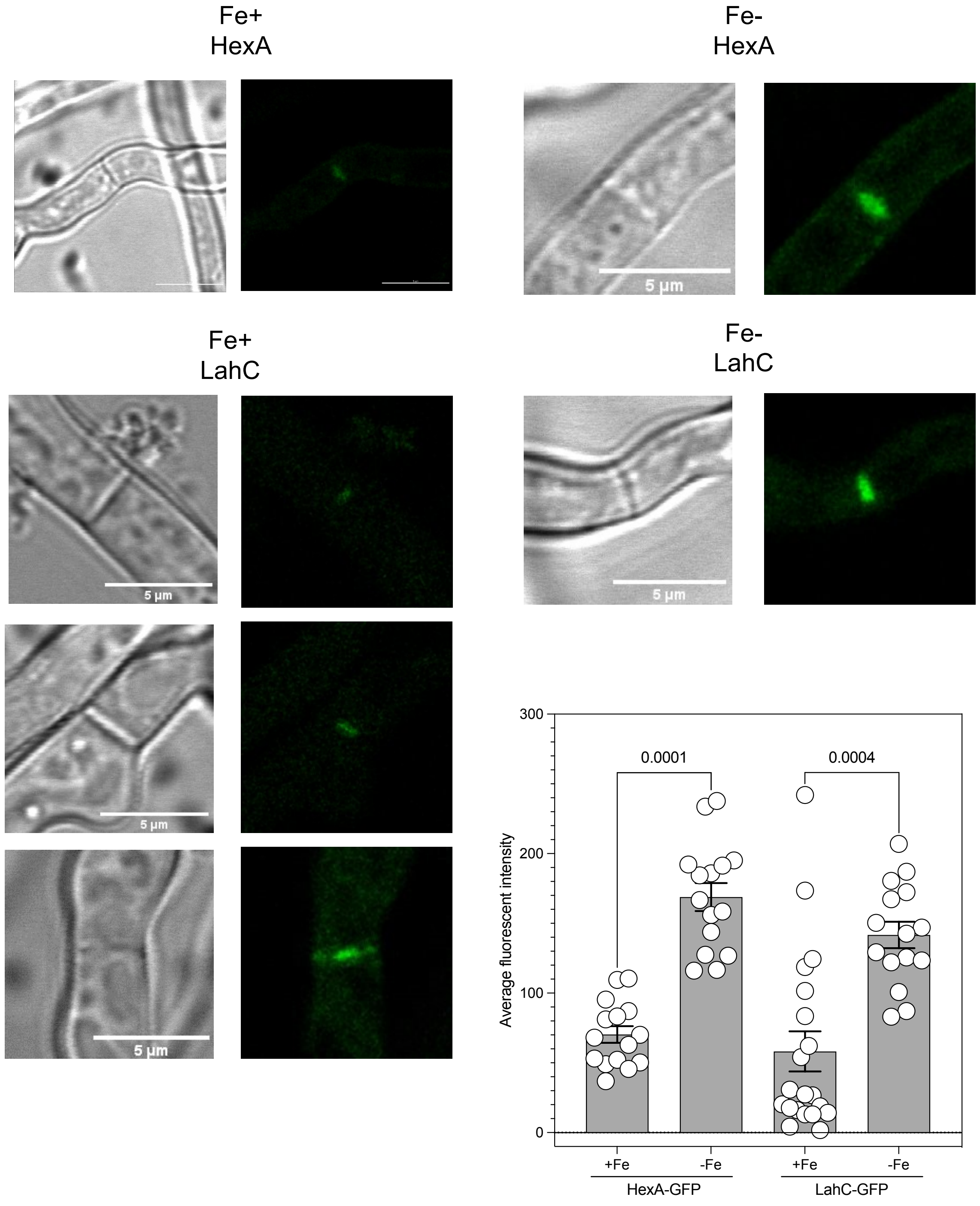

Supplemental Figure 7: HexA-GFP and LahC-GFP localisation. Microscopic analysis of localisation of HexA-GFP and LahC-GFP in individual hyphae in iron replete and limiting conditions. Fluorescent intensity was measured in ImageJ.

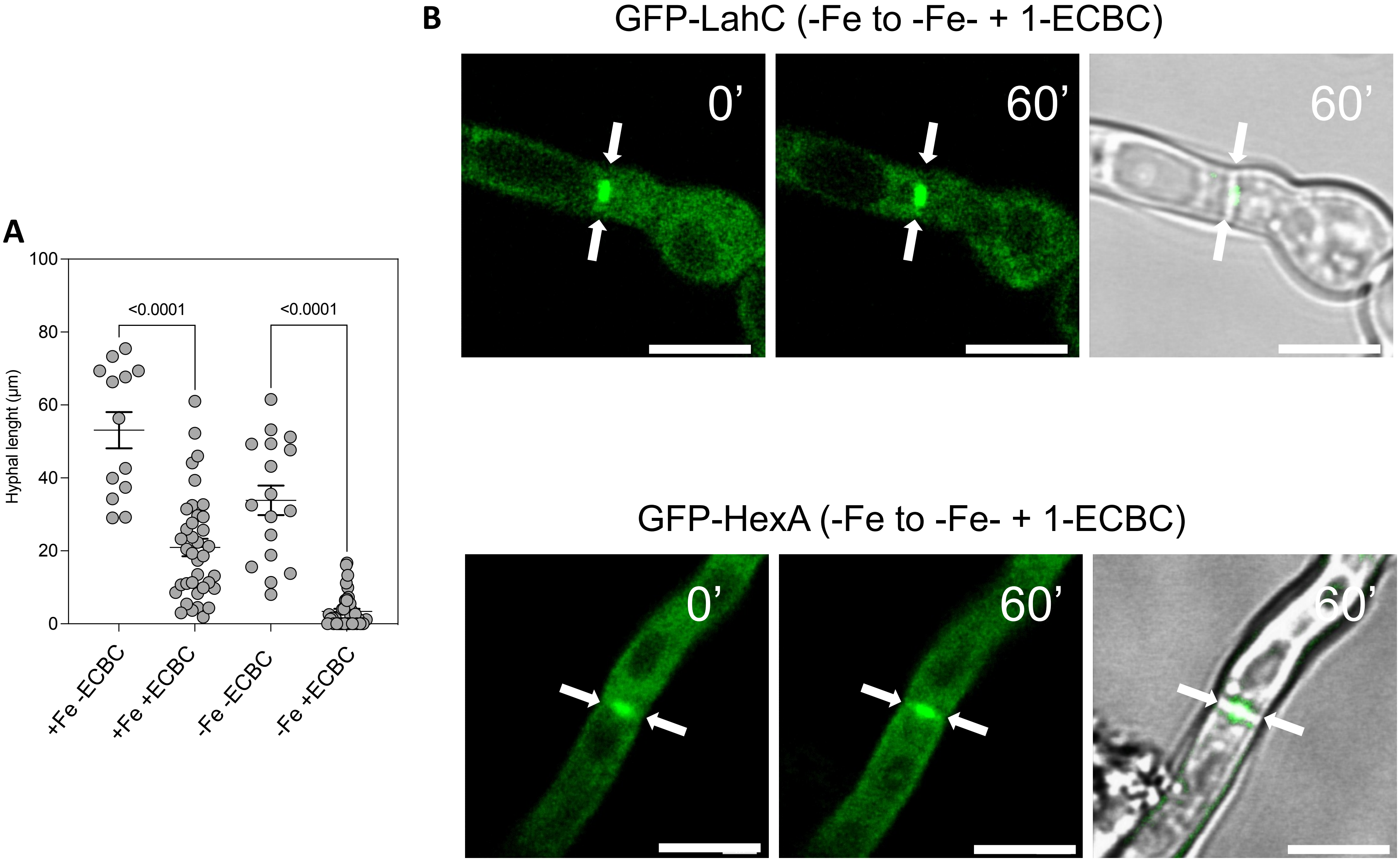

Supplemental Figure 8: GFP-LahC and GFP-HexA in response to 1-ECBC. A. hyphal length was measured in FIJI under different growth conditions and under exposure to 1-ECBC (n>12). Statistical difference was assessed by one-way ANOVA. B) Representative Microscopic images of GFP-LahC and GFP-HexA upon 1-ECBC challenge. Individual hyphae were tracked for 1 hour after the challenge.
