# Supplementary material for: Functional analysis of the Aspergillus fumigatus kinome identifies a druggable DYRK kinase that regulates septal plugging
Source: Nat Commun. 2024 Jun 11;15:4984. doi: 10.1038/s41467-024-48592-8 (PMC11166925; doi:10.1038/s41467-024-48592-8)
Supplement: Supplementary file 3 — Description of Additional Supplementary Files [file 41467_2024_48592_MOESM3_ESM.pdf]

Description of Additional Supplementary Files:

Supplementary Data 1: Overview of the kinase null collection

Tab1: Genes for which we attempted to generate null mutants in *A. fumigatus*. For those strains where homokaryotic null mutants were not generated, the results of heterokaryon rescue efforts and previous high throughput analysis of protein kinases in Aspergillus spp are given. Tab2 and 3: Outputs from Kinomer analysis of the A1163 and AF293 genomes. Tab 4: primers used in this study.

Supplementary Data 2: Relative fitness in vivo

Supplementary Data 3: Overview of phosphoproteomics data

Tab1: Quantitative abundance of proteins in A. fumigatus in the presence and absence of iron and YakA. Tab2: Relative abundance of phosphopeptides in the presence and absence of iron and YakA.

Supplementary Movie 1:

Movie showing microscopic evaluation of YakA-GFP under constant iron limitation and addition of 1-ECBC (green fluorescent channel). The stills of this video are shown in Figure 7B, lower panels. Hyphae were followed for one hour upon 1-ECBC addition and remain intact. ECBC does not induce removal of YakA-GFP once it has localised.

Supplementary Movie 2:

Movie showing microscopic evaluation of YakA-GFP under constant iron limitation and addition of 1-ECBC (bright field). The stills of this video are shown in Figure 7B, lower panels. Hyphae were followed for one hour upon 1-ECBC addition and remain intact. ECBC does not induce removal of YakA-GFP once it has localised.
