# Supplementary material for: Functional analysis of the Aspergillus fumigatus kinome identifies a druggable DYRK kinase that regulates septal plugging
Source: Nat Commun. 2024 Jun 11;15:4984. doi: 10.1038/s41467-024-48592-8 (PMC11166925; doi:10.1038/s41467-024-48592-8)
Supplement: Supplementary file 9 — Reporting Summary [file 41467_2024_48592_MOESM9_ESM.pdf]

nature portfolio

Corresponding author(s): Michael J. Bromley

Last updated by author(s): Mar 2, 2024

Reporting Summary

Nature Portfolio wishes to improve the reproducibility of the work that we publish. This form provides structure for consistency and transparency in reporting. For further information on Nature Portfolio policies, see our Editorial Policies and the Editorial Policy Checklist.

Statistics

For all statistical analyses, confirm that the following items are present in the figure legend, table legend, main text, or Methods section.

n/a	Confirmed	
☐	☒	The exact sample size (*n*) for each experimental group/condition, given as a discrete number and unit of measurement
☐	☒	A statement on whether measurements were taken from distinct samples or whether the same sample was measured repeatedly
☐	☒	The statistical test(s) used AND whether they are one- or two-sided<br>*Only common tests should be described solely by name; describe more complex techniques in the Methods section.*
☒	☐	A description of all covariates tested
☐	☒	A description of any assumptions or corrections, such as tests of normality and adjustment for multiple comparisons
☐	☒	A full description of the statistical parameters including central tendency (e.g. means) or other basic estimates (e.g. regression coefficient) AND variation (e.g. standard deviation) or associated estimates of uncertainty (e.g. confidence intervals)
☐	☒	For null hypothesis testing, the test statistic (e.g. *F*, *t*, *r*) with confidence intervals, effect sizes, degrees of freedom and *P* value noted<br>*Give P values as exact values whenever suitable.*
☒	☐	For Bayesian analysis, information on the choice of priors and Markov chain Monte Carlo settings
☒	☐	For hierarchical and complex designs, identification of the appropriate level for tests and full reporting of outcomes
☒	☐	Estimates of effect sizes (e.g. Cohen's *d*, Pearson's *r*), indicating how they were calculated

Our web collection on statistics for biologists contains articles on many of the points above.

Software and code

Policy information about availability of computer code

Data collection	The sequencing data were generated via the Illumina iSeq sequencing platform. LC-MS/MS analysis was performed on an Ultimate 3000 nano RSLC system connected to a QExactive HF mass spectrometer (both Thermo Fisher Scientific, Waltham, MA, USA). Live-cell imaging of A. fumigatus in liquid cultures was performed using a Leica TCS SP8 confocal laser-scanning microscope (Leica Microsystems Ltd., Milton Keynes, UK) equipped with photomultiplier tubes, hybrid GaAsP detectors and a 63x water immersion objective.
Data analysis	Quality control of raw reads was performed using FastQC (0.12.1), and trimmed using CutAdapt (4.9). Trimmed reads were aligned to a Fasta file containing individual barcodes using Bowtie2 (2.5.0). Counts per strain were obtained using BED IdXstats. Fitness indices were calculated using DEseq2 (1.38.3). Clustering was performed using Pheatmap (1.0.12) in Rstudio (4.2.2) with standard parameters.<br><br>The LC-MS/MS instrument was controlled by Chromeleon 7.2, QExactive HF Tune 2.8 and Xcalibur 4.0 software.<br><br>Tandem mass spectra were searched against Aspergillus fumigatus Af293 using Proteome Discoverer (PD) 2.4 (Thermo) and the algorithms of Mascot 2.4.1 (Matrix Science, UK), Sequest HT (version of PD2.4), MS Amanda 2.0, and MS Fragger 3.2.<br><br>Acquired images were analysed using Imaris v8.0 software (Bitplane Scientific software module; Zurich, Switzerland).<br><br>The structure of YakA kinase domain (Y345-I676) was determined using AlphaFold2 .<br><br>VSpipe (1.0) was used for blind docking using AutoDock Vina (1.1.2) . Druggable pockets were predicted using PockDrug , an online server which assess pocket geometry, hydrophobicity, and aromaticity. Outputs from all programmes were visualised in PyMOL 2.5.

Statistical anaysis was carried out using GraphPad PRISM 8.0.1 (La Jolla, CA, USA)

For manuscripts utilizing custom algorithms or software that are central to the research but not yet described in published literature, software must be made available to editors and reviewers. We strongly encourage code deposition in a community repository (e.g. GitHub). See the Nature Portfolio guidelines for submitting code & software for further information.

Data

Policy information about availability of data

All manuscripts must include a data availability statement. This statement should provide the following information, where applicable:

- Accession codes, unique identifiers, or web links for publicly available datasets
- A description of any restrictions on data availability
- For clinical datasets or third party data, please ensure that the statement adheres to our policy

Mass spectrometry proteomics data are available from the ProteomeXchange Consortium via the PRIDE partner repository with dataset identifier PXD042616. Source data are provided with this paper.

Tandem mass spectra were searched against the FungiDB database (2021/10/26 (YYYY/MM/DD); https://fungidb.org/common/downloads/Current_Release/AfumigatusAf293/fasta/data/FungiDB-54_AfumigatusAf293_AnnotatedProteins.fasta) of Aspergillus fumigatus Af293.

Blind docking studies were performed with the Maybridge Ro3 1000 fragment library.

Research involving human participants, their data, or biological material

Policy information about studies with human participants or human data. See also policy information about sex, gender (identity/presentation), and sexual orientation and race, ethnicity and racism.

Reporting on sex and gender	n/a
Reporting on race, ethnicity, or other socially relevant groupings	n/a
Population characteristics	n/a
Recruitment	n/a
Ethics oversight	n/a

Note that full information on the approval of the study protocol must also be provided in the manuscript.

Field-specific reporting

Please select the one below that is the best fit for your research. If you are not sure, read the appropriate sections before making your selection.

[x] Life sciences [ ] Behavioural & social sciences [ ] Ecological, evolutionary & environmental sciences

For a reference copy of the document with all sections, see nature.com/documents/nr-reporting-summary-flat.pdf

Life sciences study design

All studies must disclose on these points even when the disclosure is negative.

Sample size

For our competitive fitness analysis the read depth required has been calculated based on a minimum of 100 counts per mutant-condition in starting pools. Samples were performed in 5 replicates for our in vitro studies. As the inoculum per mouse in our in vivo studies is 5x10^5 spores, we limit the number of strains per pool to 25. Informed by pilot data from 3 independent experiments, the pooled within animal standard deviation of log transformed normalised data is 0.42. To be 80% confident (power) that a true reduction of 90% will be detected (Log2 change of 2), using a 2-sided 5% test, 5 mice are required. For in vitro competitive fitness experiments no power calculations were performed. All experiments were performed in triplicate. Statistical data were generated using DEseq2.

For our comparative virulence experiments we have performed retrospective evaluation of our data to calculate required sample sizes. 10 mice are required to detect a change in mean survival times (MST) of +2 days when compared to the wild-type/isotype control with 96% power.

No power calculations were made for any other experiments and sample sizes reflect those used in prior studies of this nature. These sample sizes were sufficient to reach statistical significance following post hoc analysis.

Data exclusions	No data were excluded from our analysis
Replication	All experiments were conducted with independent biological replicates. The number of independent replicates were consistent with similar experiments conducted within the field. All experiments had a minimum of three independent biological replicates.
Randomization	Where no randomization was applied covariates were controlled by including relevant controls in experiments. Specifically for our fitness data, known controls (wild-type or those known to have fitness defects in specific conditions) were included. Where sub pools of strains were used, strains were randomly allocated to each pool.
Blinding	Blinding and group allocations were not required or performed in this study. Data collection and analysis was performed by the same person who was not blinded to the conditions of the experiments. Biological experiments were impossible to conduct under the conditions of blinding.

Reporting for specific materials, systems and methods

We require information from authors about some types of materials, experimental systems and methods used in many studies. Here, indicate whether each material, system or method listed is relevant to your study. If you are not sure if a list item applies to your research, read the appropriate section before selecting a response.

Materials & experimental systems

n/a	Involved in the study	
☒	☐	Antibodies
☒	☐	Eukaryotic cell lines
☒	☐	Palaeontology and archaeology
☐	☒	Animals and other organisms
☒	☐	Clinical data
☒	☐	Dual use research of concern
☒	☐	Plants

Methods

n/a	Involved in the study	
☒	☐	ChIP-seq
☒	☐	Flow cytometry
☒	☐	MRI-based neuroimaging

Animals and other research organisms

Policy information about studies involving animals; ARRIVE guidelines recommended for reporting animal research, and Sex and Gender in Research

Laboratory animals	10 week old CD1 male mice (27-37g) (Charles River UK, Ltd.) were used in this study. Mice were housed in groups of 3–4 in IVC cages with access to food and water ad libitum.<br><br>We abide by the code of practice https://www.gov.uk/guidance/animal-research-technical-advice#code-of-practice-for-the-care-and-accommodation-of-animals. Mice are maintained on a 12 hour light:dark cycle, at 21 °C plus or minus 2 degrees. Relative humidity is maintained between 45 and 65%.
Wild animals	The study did not involve wild animals
Reporting on sex	Sex was not considered in study design. There is no strong evidence of a sex-bias in the frequency of aspergillosis in humans (PMID:35484713) and no discernible effect on cytokine secretion between human male and female subjects (PMID:34850023).
Field-collected samples	The study did not include field collected samples.
Ethics oversight	The mouse infection experiments were performed under UK Home office Project Licence PDF8402B7 and approved by the University of Manchester Ethics Committee.

Note that full information on the approval of the study protocol must also be provided in the manuscript.

Plants

Seed stocks	n/a
Novel plant genotypes	n/a
Authentication	n/a
